# Knocking-down of CREPT prohibits the progression of oral squamous cell carcinoma and suppresses cyclin D1 and c-Myc expression

**DOI:** 10.1371/journal.pone.0174309

**Published:** 2017-04-03

**Authors:** Juntao Ma, Yipeng Ren, Lei Zhang, Xiangpan Kong, Tong Wang, Yueyi Shi, Rongfa Bu

**Affiliations:** 1 Department of Stomatology, Chinese PLA General Hospital, Beijing, China; 2 Department of Stomatology, The First Affiliated Hospital of Dalian Medical University, Dalian, China; 3 Department of Oral and Maxillofacial-Head and Neck Surgery, Beijing Stomatological Hospital, Capital Medical University, Beijing, China; 4 School of Medicine, Nankai University, Tianjin, China; University of South Alabama Mitchell Cancer Institute, UNITED STATES

## Abstract

**Background:**

As a regulator essential for many cell cycle-related proteins, the robust expression of Cell cycle-Related and Expression-elevated Protein in Tumor (CREPT) implicates a poor diagnosis of endoderm and mesoderm-derived tumors. Whether CREPT plays the same role in the tumorigenesis derived from ectodermal tissues remains elusive.

**Methods:**

To explore the role of CREPT in ectoderm-derived tumors, cells from 7oral squamous cell carcinoma (OSCC) lines and 84clinical OSCC samples were exploited in this study. Quantitative PCR, Western blot assay and immunohistochemistry were applied in the evaluation of CREPT, cyclin D1 and c-Myc expression. Knocking-down of CREPT was performed by lentivirus delivering specific shRNA of CREPT. The effects of CREPT on OSCC were examined by cell proliferation, colony formation, apoptosis, cell migration and xenograft implantation experiments.

**Results:**

Compared with human normal oral keratinocytes, OSCC cell lines showed a significantly elevated expression of CREPT in both mRNA and protein levels. Consistently, samples from OSCC patients also exhibited a noticeably stronger CREPT expression than the noncancerous samples. In contrast, knocking down of CREPT in OSCC cell lines significantly reduced proliferation, colony formation and migration as well as the expression of cyclin D1 and c-Myc, but promoted apoptosis. Statistical analysis also suggested that CREPT expression was significantly correlated with the T and N classification of OSCC. Furthermore, CAL27 mouse xenograft model confirmed that down-regulation of CREPT prohibited cyclin D1 and c-Myc expression, through which decreased the *in vivo* tumor growth, but increased the survival ratio of hosts.

**Conclusion:**

In OSCC cell lines, up-regulated CREPT expression enhanced cell proliferation, migration and cell cycle as well as promoted cyclin D1 and c-Myc expression as it did in endoderm and mesoderm-origin tumors. Our study strongly suggests that CREPT could be used as a marker for the OSCC prognosis and might work as a potential target in future OSCC therapy.

## Introduction

As the most widely spread oral tumor, OSCC accounts for more than 90% of oral neoplasms and affects about 270,000 people throughout the world. Although a lot of advanced approaches were adopted in the therapy, there were poor improvements in the 5-year survival rate of OSCC patients [1–3]. In the last three decades, although many studies revealed that lots of risk factors, such as human papillomavirus infection and consumption of alcohol, tobacco and betel nut involved in the initiation and development of OSCC [4, 5], the genetic mechanisms triggered by these risk factors and contributing to the tumorigenesis of OSCC have not been studies thoroughly, which at least partially results in the poor prognosis and diagnosis of OSCC.

CREPT was first reported in 2012 as an accelerator of tumorigenesis due to its enhancement on cyclin D1 expression [6]. CREPT not only directly activates cyclin D1 promoter, but also can facilitate the recycling of RNA polymerase by promoting chromatin loop formation, which in turn, prevented transcription from terminating [6, 7, 8]. Similar to cyclin D1, the expression of a series of cell cycle-related factors, such as cyclin E, CDK2, CDK4 and CKD6, are also promoted by CREPT in the same manner [9]. Additionally, recent studies also revealed that CREPT could stabilize the binding of β-catenin/TCF4 complex to the promoters of cyclin D1 and c-Myc, which enforced the effects of Wnt canonical signaling on cell cycle [10]. Besides the enhancement on tumorigenesis, CREPT also plays a critical role in the activation of peripheral T cells, keratinocyte differentiation and metastasis by regulating cell cycle-related genes [11]. Since all these effector genes down-stream to CREPT also participate in a wide array of biological processes, the interference on CREPT expression is suggested to be an approach regulating multiple cell behaviors and functions [12–15].

The up-regulated CREPT expression has been detected in the carcinoma derived from mesodermal and endodermal tissues, and correlated with their poor prognosis. In colon cancer, endometrial cancer and retriperitoneal leiomyosarcoma, elevated CREPT expression is correlated with the poor differentiation, increased invasion and metastasis [6, 16, 17, 18]. In contrast, latest study indicated that siRNA-induced knocking down of CREPT significantly reduced the proliferation and migration of non-small cell lung cancer, in which the expression of c-Myc and CDC25A also decreased [19]. However, even though the increasing reports supported that elevated CREPT expression could be regarded as a marker for poor prognosis of mesoderm and endoderm-derived carcinomas, the roles of endogenous CREPT in the ectoderm-derived tumors, especially OSCC, have not been clarified. If the CREPT expression exerts the identical function in OSCC as in other tumors is the preferential question to be answered. Thus, this study was designed to investigate the role of CREPT during the progression of OSCC.

## Materials and methods

### Ethics statement

This study was approved by the Ethical Committee of Chinese PLA (People’s Liberation Army) General Hospital and conducted in accordance with the guidelines of the Declaration of Helsinki. We explained the nature and aims of the research to all subjects. All of the patients participated in this study provided written informed consent for the use of their medical records and tissue specimens for research purposes. We directly interviewed with or phoned the patientsor their relatives to collect the follow-up data.

### Samples collection

84 specimens from OSCC patients and 10 specimens from non-cancerous patients were collected in surgeries in the Chinese PLA general Hospital from Mar 2011 to Mar 2014. Normal mucosa adjacent to the surgical sites of non-cancerous patients was collected as control samples. None of the patients received preoperative therapy before surgical resection. The OSCC diagnosis was histopathologically confirmed and staged according to the 2009 UICC-TNM Classification of Malignant Tumors.

### Cell lines and cell culture

The human OSCC cell lines used in this study were HN6, OSC4, SCC15, SCC25 and CAL27. The human gastric adenocarcinoma cell line (MGC803) expressing CREPT stably was applied as positive control [6], and the normal human oral keratinocyte cell line (HOK) as normal control[10, 20, 21]. SCC25, CAL27 and HOK were obtained from the American type culture collection (ATCC). HN6 and SCC15 were kindly provided by Beijing Institute of Dental Research, Stomatological Hospital and School of Stomatology.MGC803 and OSC4 weregifted by Department of Gastroenterology & Hepatology, Chinese PLA General Hospital. The HN6, SCC15 and CAL27 cells were incubated in DMEM medium (Hyclone, Logan, UT) containing 10% fetal bovine serum (FBS; Gibco, USA). SCC25 cells were grown in DMEM/Ham’s F-12 medium supplemented with 20% FBS (HyClone, Logan, USA), hydrocortisone (40 ng/mL) and sodium pyruvate (1 mM). The OSC4 and MGC803 cells were incubated in PRMI-1640 (Gibco, USA) containing 10% FBS (Gibco, USA). The HOK cells were incubated in oral keratinocyte medium (OKM; ScienCell Research Laboratories, Carlsbad, CA.USA) containing growth supplements as previously described [21, 22].

### Quantitative PCR

Total RNA was extracted from cells by using trizol total RNA reagent (Invitrogen, Carlsbad, CA). Primers were synthesized by Sheng Gong Inc (Shanghai, China), and their sequences are shown as follow: CREPT: forward, 5'- CACGCGGGACCCATCGTCTC—3' and reverse, 5'-AGCCTTCATCTGCCTCTCTGGCA-3'; β-actin: forward, 5'-CCACTGGCATCGTGATGGA-3' and reverse, 5'-CGCTCGGTGAGGATCTTCAT- 3'[10]. Quantitative PCR (q-PCR) was performed by using the SYBR primescript RT-PCR kit (Takara, Ohtsu, Japan) in an Applied Biosystems 7500 Fluorescent Quantitative PCR System (Applied Biosystems, Foster City, CA). The reaction mixtures were incubated at95°C for 30s, followed by 33 amplification cycles of95°C for 5s and 60°C for 32s. The comparative Ct method was used to quantify the relative expression of mRNA. The expression level of β-actin was used to normalize gene-of-interest expression.

### Western blotting

Cells and xenograft tumors were washed twice with chilled PBS and homogenized directly by incubating with RIPA lysis buffer supplemented with protease inhibitors (Roche) after 48h of post-transfection. Equal amounts of proteins were size-fractionated on 4–12% SDS-PAGE gels inMOPS/SDS running buffer (Invitrogen), and transferred onto a nitrocellulose membranes (Whatman) in NuPAGE transfer buffer (Invitrogen) containing 10% methanol. Membranes were blocked with 5% skimmed milk at room temperaturefor 2h, and then incubated overnight at 4^°^C with mouse anti-human CREPT antibody (1:500 dilution in TBS-T; provided by Pro. Zhijie Changat the State Key Laboratory of Biomembrane and Membrane Biotechnology in Tsinghua University) [6, 10], anti-cyclin D1antibody (rabbit IgG, 1:1000 dilution in TBS-T), anti-c-Myc antibody (rabbit IgG, 1:1000 dilution in TBS-T; Cell Signaling Technology, Beverly, CA), and anti-β-actin (mouse IgG, 1:2000 dilution in TBS-T; ZSGB-BIO, Beijing, China), respectively. After incubation with primary antibodies, the membrane was incubated with a secondary anti-rabbit or anti-mouse IgG conjugated with horseradish peroxidase (ZSGB-BIOBeijing, China) for 1 hour at room temperature. The blots were visualized via enhanced chemiluminescence (ECL) according to the manufacturer’s protocol. Films were scanned and quantified by using a mage analysis system (Media Cybernetics, Singapore).

### Immunohistochemistry

The paraffin-embedded tissues were sliced into 4 μm sections. The sections were heated in a microwave oven for antigen retrieval, and a standard streptavidin/peroxidase complex method (SP) was used for immunostaining as previously described [23]. The anti-CREPT antibody (1:60, as mentioned above) was used as primary antibody [8, 10]. After counterstained with Meyer’s hematoxylin, the sections were observed under a light microscope. Five random fields from each section were viewed under a light microscope (Leica DM-4000B, Germany) at 400 magnification. All immunohistochemical results were examined in a blinded manner without any knowledge of the clinicopathological parameters or patient outcomes. The immunoreactivity for CREPT was recorded as strong or weak based on the staining intensity score and the percentage score. The proportion score was assigned according to the percentage of the tumor cells displaying positive nuclear staining (0: < 10%; 1: 11–30%; 2: 31–80%; 3: > 80%). The intensity score was assigned according to the average intensity of immunopositive tumor cells (0: none; 1: weak; 2: moderate; 3: strong). The expression score was calculated according to the percentage and intensity scores, which ranged from 0 to 9. The expression levels were categorized as negative (score 0), 1+ (score 1–3), 2+ (score 4–6) and 3+ (score 7–9). Any positive expression level (from 1+ to 3+) was regarded as positive expression.

### Stable transfection and selection

Cells were transfected with the lentivirus vector pLKO-puro encoding shCREPT orshcontrol (con) (Lentivirus vector pLKO-puro encoding shCREPT or shcontrol is constructed by Pro. Zhijie Chang at the State Key Laboratory of Biomembrane and Membrane Biotechnology in Tsinghua University). The shRNA sequences were as follows: shcontrol: 5’-GCGCGATAGCGCTAATAATTT-3’,shCREPT:5’-GCACGAAG ATTAGGTGCATTT-3’. SCC25 and CAL27 were seeded into 24-well plates and allowed to grow to 80% confluence. Then, medium containing shCREPT or shcontrol lentiviral particles supplied with Polybrene (8ug/ml, sc-134220, Santa Cruz, CA, USA) was added to these cells. After 12h, the original medium was replaced with fresh complete medium and the cells were subjected to the selection of stable clones in the presence of Puromycine dihydrochloride (1ug/ml, sigma) after 72h of transfection. The expression of CREPT was determined by q-PCR and Western blot as described above after 21 days of puromycin selection.

### Cell proliferation assay

For cell proliferation assay, OSCC cell lines were seeded in 96-well plates at 2×10^3^ cells/well with complete medium. Cell Counting Kit-8 (CCK-8) was used to assess cell proliferation rate at each time point according to the manufacturer’s protocol (Dojindo, Kyushu, Japan). Absorption peak at 450 nm was measured by microplate reader (Thermo Multiskan MK3, MA, USA). The experiments were independently repeated for three times.

### Colony formation assay

For the clonogenic assay, a total of 1x10^3^ cells/well were plated into 6-well plates with complete medium. After being cultured for 2 weeks, the cells were fixed with methanol for 10 min, followed with 0.1% crystal violet staining for 10 min. The colony number was counted under microscope. The experiments were independently repeated for three times.

### Migration assay

For cell migration assay, shCREPT- and con-treated cells were seeded in a 6-well plate with complete medium until they reached a confluent monolayer. Scratches with the width of 500 μm were created in the middle of each well by using a micropipette tip. The plate was incubated at 37°C with 5%CO2 atmosphere. The results were visualized by measuring the scratch width. The experiments were independently repeated for three times.

### Apoptosis assay

To analyze the effects of CREPT on apoptosis, sh-CREPT and con-treated cells were harvested, washed twice and re-suspended in ice-cold PBS. 1×10^6^ cells were incubated in 100 μL binding buffer containing 5μL Annexin V-FITC at 37°C in darkness for 15 min. The resulting cells were analyzed by flow cytometer (Beckman Coulter, USA).

### OSCC xenograft tumors

All animal experiments were approved by the Animal Research Committee at Beijing Laboratory Animal Research Center and were carried out in accordance with established International Guiding Principles for Animal Research. The authorized Protocol Number is P2015015. CAL27con and CAL27shCREPT treated cells (4×10^6^ cells/mouse) were subcutaneously injected into the right flank of to 5-week old female athymic nude mice to form xenograft tumors. Twenty mice were used for each group. After 28 days, when mice were sacrificed by cervical dislocation, tumors were harvested for Western blot analysis.

### Statistical analysis

The data were analyzed using SPSS 17.0 software (IBM, NY, USA). The Mann-Whitney *U*test, the Student’s t-test, the X^2^ test, the Fisher’s exact test and the Kaplan-Meier(log-rank test) were used in this study. p< 0.05 was regarded as a statistically significant difference.

## Results

### CREPT expression was significantly up-regulated in OSCC cell lines and OSCC samples

**Fig 1:**
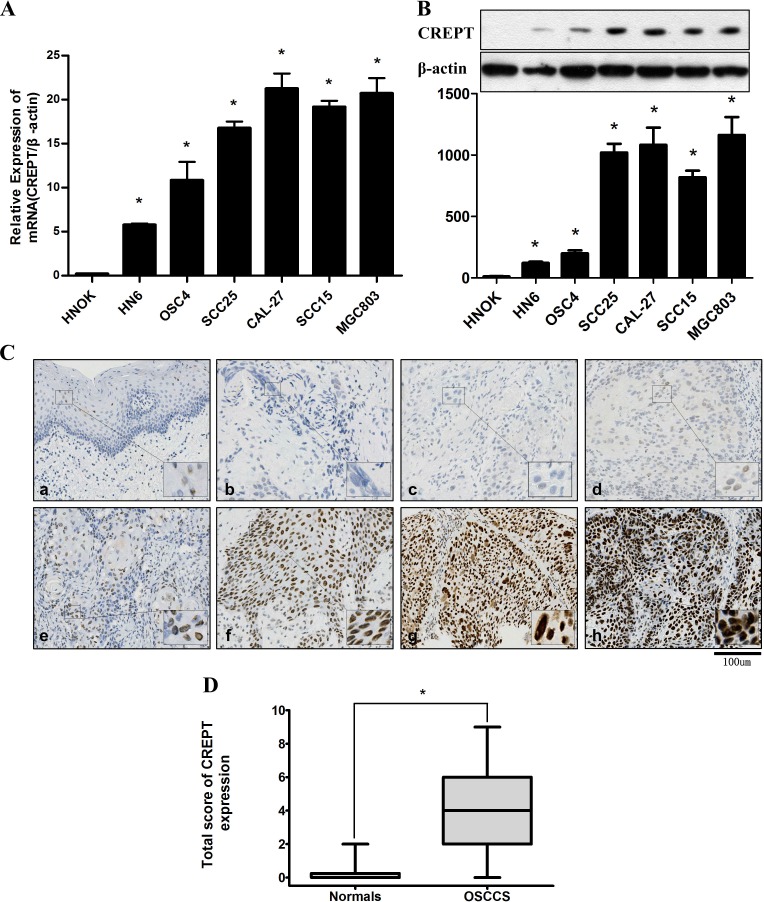
Expression levels of CREPT in OSCC cell lines and OSCC samples. **(A)** CREPT mRNA is up-regulated in all 5 OSCC cell lines and MGC803, compared with the HOK, showed by q-PCR analysis. (*p<0.05, Mann-Whitney *U* test). (B) Western blotting analysis showed that the CREPT protein level was up-regulated nearly in allcell lines, compared with the HOK. Densitometric CREPT protein data were normalized to the β-actin protein levels. (C) IHC of CREPT on primary OSCC samples. (a) Negative CREPT expression in normal oral tissue; (b, c) Negative expression of CREPT in OSCC tissues; (d, e) Weakly positive expression of CREPT in OSCC tissues; (f, g, h) Positive and strong positive expression of CREPT in OSCC tissues (Original magnification is 200 fold, bar = 100 μm); (D) Box showed that the statistical comparison of CREPT protein levels in OSCC and normal oral tissues (*p<0.05, Mann-Whitney *U* test).

To verify if the CREPT expression is elevated in OSCC as in other tumors, q-PCR and Western blotting were performed in OSCC cell lines (HN6, OSC4, SCC25, CAL27 and SCC15), MGC803 and HOK to assess CREPT levels. CREPT mRNA was remarkably elevated in OSCC cell lines as in MGC803, compared with the HOK (Fig 1A; S1 File). Consistently, the protein level of CREPT in OSCC cell lines and MGC803 was noticeably higher than that in HOK(Fig 1B; S1 File). To further confirm the *in vitro* result, the CREPT expression in 84 paraffin-embedded OSCC samples and 10 non-cancerous oral mucosa were evaluated by immunohistochemical staining (Table 1;Table 2; Fig 1C). The immunohistochemical scores ranged from 0 to 9 in the OSCC samples (median = 3.1714), but from 0 to 2 in the normal counterparts (median = 0.3000). Moreover, we found that the average CREPT expression in OSCC samples was relatively higher than that in non-cancerous samples, but the statistical significance was denied (Fig 1D; S1 File). Thus, we further analyzed the relationship between clinicopathologic characteristics and CREPT expression levels in OSCC individuals (Table 1). Although there was no significant correlation of CREPT levels between patients’ age, gender, smoking status and alcohol, and the location, differentiation and stage of OSCC, the elevated expression level of CREPT was markedly correlated with the larger tumor size (T classification) and more lymph-node metastasis (N classification).

**Table 1:** Correlation between CREPT expression and clinical classification in OSCC.

Parameter	Result of immunostaining						
	No. of patients/ (%)						
	Total	CREPT(-)		CREPT(+)		p value	
Age at surgery (year)							
<60	35	11	31%	24	69%	0.059	
≧60	49	7	14%	42	86%		
Gender							
Male	63	10	16%	53	84%	0.061	
Female	21	8	38%	13	62%		
Smoking							
Yes	58	14	24%	44	76%	0.366	
No	26	4	15%	22	85%		
Alcohol							
Yes	30	8	27%	22	73%	0.383	
No	54	10	19%	44	81%		
T-primary tumor							
1+2	49	16	33%	33	67%	0.003*	
3+4	35	2	6%	33	94%		
N-regional lymph node							
-	44	14	32%	30	68%	0.015*	
+	40	4	10%	36	90%		
Stage							
Ⅰ+Ⅱ	39	12	31%	27	69%	0.052	
Ⅲ+Ⅳ	45	6	13%	39	87%		
Histopathologic type							
Well	45	7	16%	38	84%	0.370	
Moderate	25	7	28%	18	72%		
Poor	14	4	29%	10	71%		
Tumor site							
Tongue	31	5	16%	26	84%	0.854	
Gingiva	18	4	22%	14	78%		
Oral floor	17	5	29%	12	71%		
Buccal mucosa	12	3	25%	9	75%		
Soft palate	6	1	17%	5	83%		

*p values are obtained from X^2^ test, For Gender p values are obtained from Fisher’s exact test, significant difference, p< 0.05

**Table 2:** CREPT expression in non-cancerous specimens from 10 patients.

Parameter	Result of immunostaining				
	No. of patients/ (%)				
	Total	CREPT(-)		CREPT(+)	
Age at surgery (year)					
<30	6	6	100%	0	0%
≧30	4	3	75%	1	25%
Gender					
Male	6	6	100%	0	84%
Female	4	3	75%	1	25%
Smoking					
Yes	3	3	100%	0	76%
No	7	6	83%	1	17%
Alcohol					
Yes	1	1	100%	0	0%
No	9	8	89%	1	11%
SampleCollection site					
Tongue	1	1	100%	0	0%
Gingiva	5	5	100%	0	78%
Buccal mucosa	4	3	75%	1	25%

10 specimens were healthy oral mucosal or epithelial specimens collected from patients aged22–35, undergoing extraction of tooth and mucinous glandular surgery.

### Down-regulated CREPT expression decreased *in vitro* proliferation, colony formation, cell survival and migration in OSCC cells

**Fig 2:**
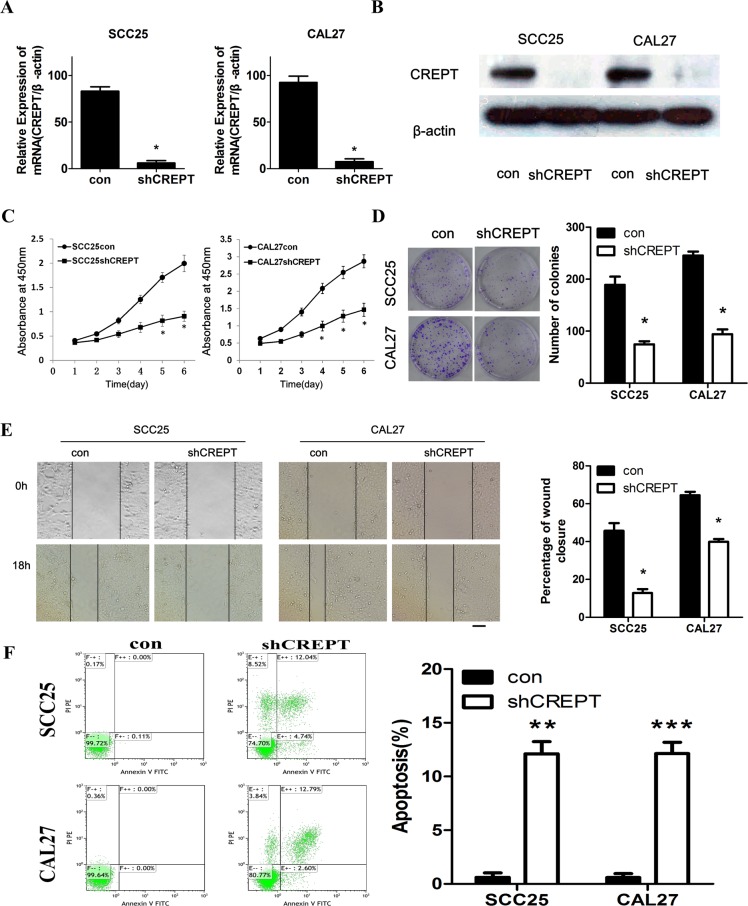
Effects of CREPT knocking-down on cell proliferation, colony formation, apoptosis and migration in OSCC cell lines. (A) CREPT transcription in shCREPT-transfected cells was significantly lower than that in the con-transfected cells (*p<0.05, Mann-Whitney *U* test). (B) The amount of CREPT protein in shCREPT-transfected cells was decreased markedly compared with the con-transfected cells. (C) Growth kinetics analysis showing that SCC25 and CAL27 cells with shCREPT transfection had lower proliferationcapability in comparison with control cells. (*p< 0.05, Mann-Whitney *U* test). (D) The number of colonies in SCC25 and CAL27 shCREPT-transfected cells were obviously decreased compared with control groups (*p<0.05, Mann-Whitney *U* test). (E) Migratory assay of shCREPT cells. To evaluate the effect of CREPT knock-down on migration, uniform scratches were made in confluent layers of the shCREPT- and con-transfected SCC25 and CAL27cells.The extent of closure was monitored visually every 6 hours for 24 hours. The mean value was calculated from data obtained from three separate chambers. The scratch width had decreased significantly in the culture of control cells after 18 hr, whereas there was still a wider gap in the shCREPT cells. (F) Flow cytometer assay show that Annexin V expression was significantly increased to 13.09% (SD = 3.39%, p<0.01) and 13.34% (SD = 1.92%, p<0.001) in the CREPT-shRNA SCC25 and CAL27 groups, respectively, from 0.93% (SD = 1.15%) and 0.33% (SD = 0.43%) inthe control groups. (*p< 0.05,* * p<0.01 and *** p<0.001 in T-test &Mann-Whitney *U* test; Original magnification is 100 fold. Scale bars, 100μm).

Taking the advantages of CREPT shRNA lentiviral particles, knocking down of CREPT was able to be performed *in vitro*. After the stable transfection of CREPT shRNA (shCREPT) and the control shRNA (con) into SCC25 and CAL27 cells, q-PCR analysis revealed that the CREPT mRNA levels in shCREPT transfected cells were significantly lower than those in the con-transfected cells(Fig 2A; S2 File). The validation of CREPT knock-down was further confirmed by Western blots, in which CREPT expression were almost diminished in shCREPT-transfected SCC25 and CAL27 cells (Fig 2B). To clarify the roles of CREPT in OSCC cell lines, we monitored cell proliferation for 6daystoaddress the effect of CREPT on proliferation. The shCREPT-transfected SCC25 and CAL27 cells decreases approximately 57% and 45%, respectively, in proliferation, compared with the con-transfected cells after 6days (Fig 2C; S2 File). Colony formation assay was used to measure tumorigenic ability *in vitro*, exhibiting an approximately 2.6 and 2.4 folds lower in the colony number of CREPT-shRNA transfected cells than that of control shRNA transfected counterparts (Fig 2D; S2 File). Cell migration assay was performed to study the influence of CREPT on metastatic capability. After the uniform scratches were made in the confluent cell layers, they closed later in the shCREPT-transfected cells than in the con-transfected cells in both OSCC cell lines (Fig 2E; S2 File). Consistent with the enhanced proliferation, further investigation on Annexin V expression showed that when the CREPT expression was suppressed, the Annexin V positive ratios in both SCC25 and CAL27 lines were greatly increased (Fig 2F; S2 File), indicating an increased apoptosis due to CREPT suppression. In summary, knocking down of CREPT expression decreased cell proliferation, colony forming and migration capabilities, but promoted cell death in OSCC cell lines.

### Knocking-down of CREPT suppressed cyclin D1 and c-Myc expression in OSCC cell lines

**Fig 3:**
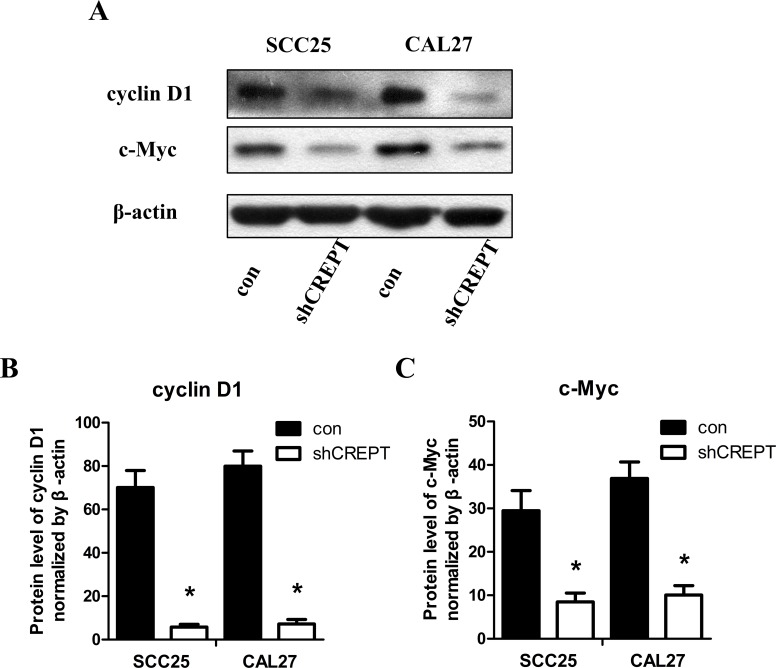
Knocking-down of CREPT down-regulated cyclin D1 and c-Myc expression in OSCC-derived cell lines. **(A)** The cyclin D1 and c-Myc levels in con- or shCREPT-treated cells as determined by Western blotting with β-actin as the loading control. (B) Statistics showed that the amounts of cyclin D1 and c-Myc protein in shCREPT-transfected cells were decreased markedly compared with the con-transfected cells. (*p <0.05, Mann-Whitney *U* test). (C) Statistics showed that the cyclin D1 and c-Myc protein expression in shCREPT-transfected cells were decreased markedly compared with the con-transfected cells. (*p <0.05, Mann-Whitney *U* test).

Extensive studies disclosed that cyclin D1 and c-Myc played important roles in regulating proliferation and migration in OSCC cells [10, 12–15]. Since previous studies also revealed that CREPT directly enhanced the expression of cyclin D1 and affected c-Myc expression [6, 10], we focused our attention to establish the relationship between the expression of CREPT and cyclin D1 as well as c-Myc in OSCC. Western blot assay showed that when SCC25 or CAL27 cells were transfected by CREPT-shRNA to knock down the endogenous CREPT expression, the amounts of cyclin D1 and c-Myc proteins also exhibited the noticeable decreases (Fig 3A). The statistical analysis indicated that in CREPT-shRNA transfected SCC25 or CAL27 cells, both the cyclin D1 and c-Mycexpression were dramatically decreased, compared with their con-transfected groups (Fig 3B and 3C; S3 File).

### Knocking-down of CREPT increased host survival rate and suppresses OSCC tumor weight *in vivo*

**Fig 4:**
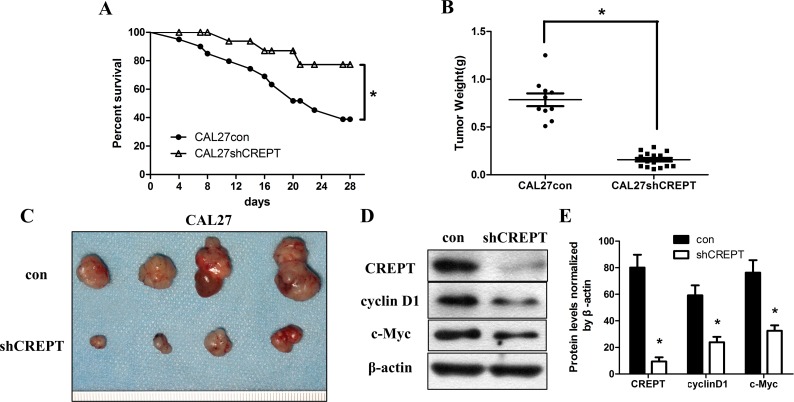
CREPT knocking-down increased survival rate and reduced tumor volume *in vivo*. (A) Kaplan–Meier plots of survival analysis of the SCC25 and CAL27 experimental groups compared with their control groups (*p< 0.05, log-rank test). (B) Tumor weights were measured after 28 days(*p< 0.05, Mann-Whitney *U* test). (C) At 28 days post implantation, tumor xenografts were harvested. Representative images of tumors are shown. (D)CREPT, cyclinD1 and c-Myc proteins in tumor xenografts wereassessed by Western blotting with β-actin as the loading control. The CREPT, cyclin D1 and c-Myc proteins in shCREPT-transfected xenografts were decreased remarkably (*p< 0.05, Mann-Whitney *U* test).

To verify that CREPT plays the same role *in vivo* and *in vitro*, a xenograft tumor model was generated by injecting con- and shCREPT-treated CAL27 cells into nude mice. After 28 days of implantation, the treatment of shCREPT transfection raised the survival rate of nude mice carrying CAL27 cells to 75% from the 40% in con-treated CAL27 group (Fig 4A; S4 File). Consistent with the previous finding that CREPT stimulated cell proliferation, the xenograft tumor weights after 28 days post-implantation were significantly repressed in the shCERPT transfected CAL27 groups. The suppression on CREPT of CAL27 xenografts decreased the mean tumor weight to about 0.1581g, almost 5 folds less than those of the control xenografts (Fig 4B; S4 File). Correspondingly, the volumes of shCERPT treated CAL27 tumor xenografts were obviously smaller than those in control treated CAL27 xenografts (Fig 4C). The CREPT, cyclinD1 and c-Myc levels in tumor xenograft lysates examined by Western blot assay indicated that CREPT, cyclinD1 and c-Myc were all down-regulated by the shCREPT treatment compared to those bearing CAL27 con cells (Fig 4D). The densitometric quantification revealed that shCREPT treatment decreased intensities of the CREPT, cyclinD1 and c-Myc bands compared with those from their con-treated xenografts (Fig 4E; S4 File). These results suggested that suppression on CREPT could inhibit tumor growth and significantly raise the survival rate of OSCC patients, which might result from the repression on cyclin D1 and c-Myc expression.

## Discussion

In this study, we found that in all the OSCC cell lines and most of the clinical OSCC samples, CREPT expression is dramatically elevated. This elevation greatly enhances cell proliferation, colony formation, cell survival, migration and the expression of cyclin D1 and c-Myc. Therefore, for the first time, we verify that CREPT plays the same role in the tumorigenesis of ectoderm-derived OSCC as it does in mesoderm- and endoderm-derived tumors. Additionally, we discover that although the up-regulation of CREPT expression is not detected in all clinical OSCC samples, the elevated expression is statistically associated with the T and N classification of OSCC cases, suggesting that the expression level of CERPT can be applied into the clinical prognosis of OSCC. Most interestingly, when the CREPT expression was knocked down by siRNA, the size and weight, as well as the cyclin D1 and c-Myc expression, of OSCC xenografts were significantly decreased. Combined with the significant increase in the survival rate of the host mice by CERPT knock-down, CREPT may be considered as a predicted target for the future anticancer medicine.

Previous studies reported that CREPT was highly expressed in various mesoderm- and endoderm-derived tumors [6, 16, 17], whether CREPT was attributed to the ectoderm-derived tumors, especially the OSCC, remained to be clarified. In our present study, all the established OSCC cell lines gave rise to the robust expression of CREPT. However, the detection of CREPT expression in the majority of clinical OSCC samples, instead of all of them, suggested that CREPT was not the essential cause for the OSCC tumorigenesis. We speculated that this inconsistency on CERPT expression between OSCC cell lines and clinical samples was originated from the colony competition during cell line establishment. Since CREPT endows cells with the proliferation competence, the CREPT positive colony acquires growth advantage during the screen and finally overwhelms the CREPT negative colonies, which results in all the established OSCC cell lines with the strong CREPT expression. In contrast, tumor cells in the clinical OSCC samples did not undergo so intense colony competition as in the *in vitro* condition, which allowed the CREPT negative colonies to get a chance for expansion.

Although the CERPT expression is hard to serve as a hallmark for all OSCC, it is strongly suggested to predict the malignancy of OSCC. This notion was supported by the larger primary OSCC tumor sizes and more lymph-node metastasis in the CREPT positive group than those in the negative group (Table 1). Moreover, both the up-regulated CREPT transcription and protein level were closely associated with the degree of differentiation and the clinical stage of a variety of human malignant tumors [6, 16, 17]. Our statistical data found that the CREPT-expressing OSCC cases usually suffer from a larger tumor size and frequent lymoph node metastasis, implicating the notion that CREPT can be correlated to a poor prognosis of OSCC [23–26]. The results of OSCC xenograft implantation experiments also support this notion, because knocking-down of CREPT reduced the sizes of xenografts from the CREPT-expressing OSCC cell lines and increased the host survival ratio.

The effects of CREPT knocking-down on the behaviors of OSCC cell lines and the growth of OSCC xenografts demonstrate the enhancement of CREPT on OSCC cell proliferation, colony formation, cell survival and migration. Identical to the role of CREPT in other carcinomas, such enhancement may be also fulfilled through the activation of cyclin D1 and c-Myc [6, 10]. Cyclin D1 regulates cell cycle by controlling G1/S transition, and the duplication or up-regulation of cyclin D1 contributes to multiple cancers [27–29]. C-Myc acts as an oncogenic transcription factor, recognizing the E-box and the related sequences in the promoters of target genes. The elevation of c-Myc level markedly increases both metastasis and invasion capabilities of tumors[12–15].Although the direct activation of cyclin D1 by CREPT had been proved in colon cancer, our present studyonly verified the correlationship between the decreased expression of cyclin D1 and the knocking-down of CREPT both *in vitro* and *in vivo* cases of OSCC. We speculate that CREPT can increase the malignancy of OSCC by direct activating cyclin D1 promoter. However, if CREPT follows this proposed mechanism during the progression of OSCC requires further confirmation. Additionally, if all the effects of CREPT elevation on OSCC derived from the up-regulation of cyclin D1 and c-Mycrequires further investigation, because CREPT is also able to stabilize β-catenin/TCF4 complex to activate a wide array of target genes, including but not being limited to c-Myc [10].

In summary, our study demonstrates that CREPT expression is elevated in majority of OSCC samples and all OSCC cell lines. The elevated CREPT expression increases the cell proliferation, colony formation, cell survival and migration, which enhances the clinical malignancy of OSCC. Promoted OSCC malignancy by CREPT is correlated with the up-regulated cyclin D1 and c-Myc expression. Therefore, CREPT may work as a potential indicator for OSCC prognosis and target for anticancer medicine.

## Supporting information

S1 File The raw values of Q-PCR andblotting density of CREPT expression in the OSCC lines, and the scores of CREPT expressionin tumor samples. (RAR)

S2 File The raw vales of Q-PCR, CCK-8 absorbance, colony number, scratch width and Annexin V percentages in SCC25 and CAL27 lines after CREPT knocking-dwon. (RAR)

S3 File The raw data of blotting densities for the Cyclin D1 and c-Myc bands in SCC25 and CAL27 lines after CREPT knocking-dwon. (RAR)

S4 File The survival numbers of the nude mice implanted OSCC xenografts, the weights of OSCC xenografts and the raw vales of band densities for the Cyclin D1 and c-Myc expression in the xenografts. (RAR)
